# A qualitative study exploring healthcare transition experiences among newcomer international students in Canada and opportunities for pharmacist support

**DOI:** 10.1371/journal.pone.0356780

**Published:** 2026-08-28

**Authors:** Yasmin H. Aboelzahab, Andrea McCracken, Andrew D. Pinto, Lisa M. McCarthy, Lisa Dolovich

**Affiliations:** 1 Leslie Dan Faculty of Pharmacy, University of Toronto, Toronto, Ontario, Canada; 2 Upstream Lab, MAP Centre for Urban Health Solutions, Li Ka Shing Knowledge Institute, Unity Health Toronto, Toronto, Ontario, Canada; 3 Department of Family and Community Medicine, St. Michael’s Hospital, Toronto, Ontario, Canada; 4 Department of Family and Community Medicine, Faculty of Medicine, University of Toronto, Toronto, Ontario, Canada; 5 Division of Clinical Public Health, Dalla Lana School of Public Health, University of Toronto, Toronto, Ontario, Canada; 6 Institute for Better Health, Trillium Health Partners, Mississauga, Ontario, Canada; University of Science and Technology of Fujairah, YEMEN

## Abstract

**Background:**

Newcomer international students in Canada often face complex barriers when accessing healthcare, including limited knowledge of available services, insurance challenges, and mental health concerns. These difficulties may impact their well-being and disrupt continuity of care. Pharmacists, as accessible healthcare providers, can support these students during their healthcare transition. This study explored international students’ experiences with the Canadian healthcare system and identified pharmacy-led strategies to address care gaps.

**Methods:**

A qualitative exploratory descriptive study was conducted using virtual semi-structured interviews with 23 newcomer international students at the University of Toronto. Data were analyzed using conventional content analysis. Transition Theory was used as a framework to capture the layered nature of students’ healthcare transition.

**Results:**

Four overarching concepts and their related themes were identified. Navigating the Unknown described participants’ early confusion, emotional stress, and unfamiliarity with the Canadian healthcare system. Building Bridges reflected their efforts to adapt through self-education and community support, though these strategies were sometimes influenced by misinformation. Pharmacist as a Guide captured participants’ experiences with pharmacists who were viewed as accessible and trusted healthcare providers supporting medication management and navigating healthcare services. Lastly, Overcoming Barriers, Building Resilience, and Improving Access reflected participant-driven recommendations, including expanding pharmacists’ roles, improving health education, and offering virtual care solutions to better support international students and their families.

**Conclusion:**

International students face multiple challenges in accessing healthcare. Pharmacy-led education, culturally responsive support, and virtual care services can improve access and health outcomes. These findings highlight opportunities for system-level strategies to support this underserved population.

## 1. Introduction

Canada is recognized as one of the healthiest countries in the world [1]. However, despite ongoing efforts, variations in health status, outcomes, and access to healthcare services persist across different populations [1]. The Government of Canada emphasizes that health equity is achieved by eliminating barriers that lead to these health disparities and by ensuring access to the resources and opportunities that support quality care for all [1,2]. Among those facing limited access to the healthcare system are newcomers who have arrived in Canada within the past few years, typically less than five years [3–5].

Newcomers face several barriers including unfamiliarity with navigating and using healthcare services, cultural and traditional differences, economic challenges, and language barriers [6,7]. Such struggles can affect their health by hindering their ability to prevent or manage illnesses which may eventually lead to poorer health outcomes and reduced quality of life [3,8]. Given the large number of newcomers in Canada, these challenges can also place additional strain on the healthcare system and impact the overall health of the Canadian population [9].

International students represent a substantial part of Canada’s newcomer population, with their numbers reaching one million at the end of 2023 [10]. Attracted by the high quality of post-secondary education, many international students choose Canada as their destination [11]. They play a vital role in contributing to the country’s cultural diversity and economic development [11,12]. However, with the increasing pressure on public services including healthcare, the Canadian government has recently taken steps to manage the growing number of international students to ensure that essential supports such as housing, health services, and social resources are available to promote their well-being and academic success [12].

Many international students have limited knowledge of the Canadian healthcare system, including how to access services and understand healthcare policies [13,14]. Combined with social isolation and limited family support, this can create anxiety around becoming ill and hinder the development of effective coping strategies [13,14]. As a result, students may struggle to access quality care, impacting their overall well-being and ability to fully engage in academic and social life [14,15]. Difficulties in accessing medications and understanding prescription instructions further complicate their healthcare experience [14]. Such challenges suggest that adapting to the Canadian healthcare system is part of a broader transition experience that extends beyond knowing how to access services. Transition Theory, a middle-range theory, explains how individuals experience and respond to transitions over time as they encounter changes in life, health, roles, relationships, and environments [16,17]. Applying this lens supported understanding of how newcomer international students navigated healthcare-related changes within the wider context of adjusting to life in Canada [16,17].

The evolving role of pharmacists in Canada enables them to contribute meaningfully to patient care, especially for populations with limited healthcare access [18–20]. Through their geographic accessibility, pharmacotherapy expertise, and broad range of clinical services, pharmacists enhance medication safety and health outcomes by addressing issues such as improper dosing and inappropriate use [19,21,22]. In addition, pharmacists offer virtual care services to reach patients unable to access in-person care [18,23]. Pharmacists also have a key role in supporting care transitions between different healthcare settings to ensure continuity of care [24,25].

Therefore, pharmacists are well-positioned to support newcomer international students with limited knowledge of the Canadian healthcare system in navigating services and managing their medications [26]. This support could enhance their healthcare experiences and promote better health outcomes for this underserved population [9].

The aim of this study was to better understand the lived experiences of newcomer international students as they transition to the healthcare system in Canada. It also aimed to identify the challenges they face which can help reveal gaps in the existing support system that may disrupt continuity of care. Moreover, the study aimed to inform strategies for pharmacists to better support this population, including exploring the role of virtual care in addressing their healthcare needs.

## 2. Methods

### 2.1. Study design

The study followed an exploratory descriptive qualitative design using interviews, and was conducted as per our previously published protocol [27]. The exploratory component addressed the limited literature on newcomer international students' healthcare transition, while the descriptive aspect aimed to provide deeper insights into their lived experiences [28,29]. This approach aimed to identify practical strategies to support the healthcare needs of students and their families [28,30]. The design also supported close engagement with participants to understand how healthcare transition was shaped by personal, family, community, and healthcare system contexts [28,29].

### 2.2. Theoretical framework

**Fig 1:**
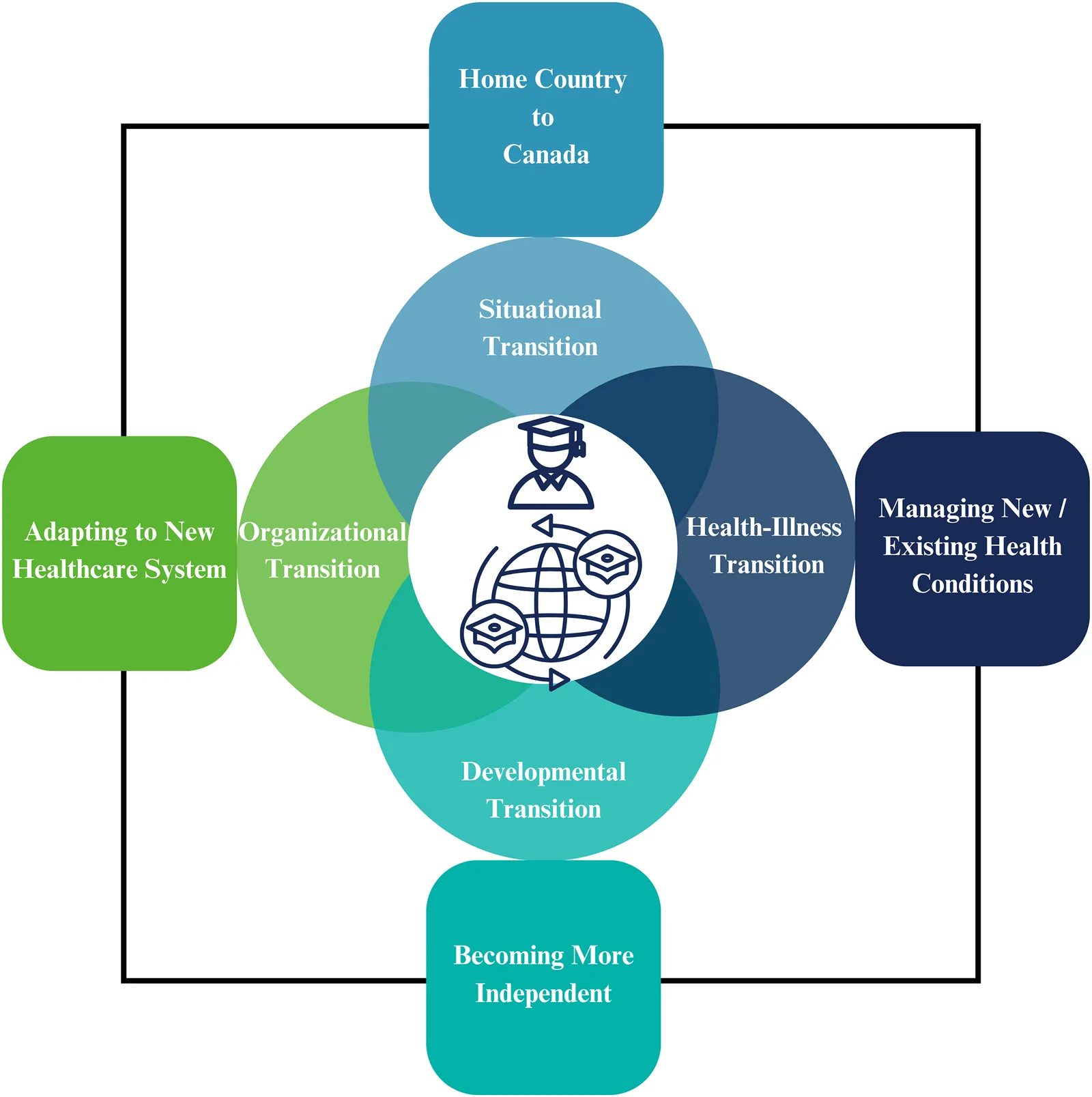
Transition Theory applied to newcomer international students’ healthcare experiences, illustrating situational, developmental, organizational, and health-illness transitions [16].

Drawing upon Transition Theory by Meleis, this study explored the dynamic processes involved in the healthcare transition of newcomer international students as they navigated the Canadian healthcare system [16,17]. Transition Theory distinguishes between different types of transitions: developmental, situational, health–illness, and organizational [16,17] (Fig 1).

It also provided a comprehensive framework for understanding the complex psychological, social, and cultural dimensions that shape students’ adaptation from initial entry to eventual integration into a new healthcare system [16,17]. This theoretical lens supported an in-depth exploration of international students’ challenges, coping strategies, and healthcare needs, highlighting the critical role of support systems in adaptation and informing how pharmacy services could be tailored to address their unique healthcare barriers [16].

### 2.3. Recruitment

Participants were recruited using purposeful sampling through various channels including displaying the study poster at Discovery Pharmacy at the University of Toronto (U of T), as well as sharing a digital version through international student advocacy groups and relevant social media platforms. Participants received a $20 gift card as a token of appreciation for their time. Participants were eligible if they were U of T international students, currently residing in Ontario, aged 18 years or older, had arrived in Canada more than one month and less than five years before participation, and were able to communicate in English using phone or internet-based platforms. Students who had a Canadian partner were excluded because they might have more direct access to informal guidance about Canadian healthcare services through their partner. International student status and other eligibility criteria were assessed through self-reported screening information. Participants’ affiliation with U of T was supported by university-based recruitment channels and communication using their university email addresses.

Recruitment was conducted through both pharmacy-based and broader university-based channels to include students with different levels of connection to healthcare and pharmacy services. This approach was intended to capture a range of experiences, including students who had already interacted with healthcare services and those who may have been less connected to existing support. Students were also from diverse countries of origin to reflect different prior healthcare system experiences and support richer insights into common healthcare transition challenges [31]. Recruitment took place between September 1, 2023, and April 18, 2024.

### 2.4. Data collection

Data collection was carried out using a semi-structured interview guide, which refers to the pre-developed set of open-ended interview questions and probes used to guide the participant interviews [32]. The guide was developed, reviewed by the study investigators, and refined through team reflections to enhance clarity and better align with the study’s objectives.

The interview guide was pilot-tested with three participants who were newcomer international students recruited through the same study pathways and eligibility criteria as other study participants. The pilot interviews were included in the final sample. Pilot testing was used to assess the flow, interpretation, and relevance of the questions, particularly because participants’ first language may not have been English. Pilot feedback helped determine whether questions were understood as intended and whether additional probes or wording changes were needed. Based on this process, minor refinements were made to improve clarity and reduce ambiguity in the wording of questions and probes (Refer to S1 Appendix for the updated interview guide, with refined or added questions underlined).

During interviews, the interviewer used probes and clarifying questions to confirm the context of participants’ responses, including whether they were describing experiences in Canada or in their countries of origin. This approach helped ensure that participants’ responses were interpreted within the intended healthcare transition context.

Virtual interviews were conducted via Microsoft Teams, which was also used for recording and transcription. Thematic saturation was reached as the data indicated shared participant experiences and no additional themes relevant to the research objectives [33]. Data collection and preliminary analysis were conducted concurrently to support ongoing assessment of the richness and adequacy of the data. Saturation was assessed through observing repeated patterns across participants’ responses, redundancy in information related to the study objectives, and increasing stability of the coding framework [34,35]. Newly collected data were compared with earlier interviews to assess whether additional interviews were contributing new insights or mainly deepening and confirming existing categories and concepts. Given the focused aim of the study, the specificity of the sample, and the richness of the data generated through semi-structured interviews, data collection was finalized after 23 interviews when the dataset was determined to be sufficient to address the study objectives.

All participants were provided with information about the study, its purpose, and what participation would involve. They consented to participate in the study by signing the written consent using the Research Electronic Data Capture (REDCap) platform. Participants were reminded that participation was voluntary and that they could skip any question during the interview. To help participants feel informed and comfortable, interviews began with an introduction to the study and an opportunity for participants to ask questions. They were also made aware that demographic information was collected at the beginning of the interview to confirm their eligibility and contextualize their later responses, including the timing of their healthcare transition. Interviews lasted between 30-60 minutes.

### 2.5. Data analysis

Conventional content analysis was conducted to systematically analyze the data [36]. The interviews were transcribed, and open inductive coding was used to capture key ideas, allowing themes and concepts to be identified directly from the transcripts [36,37]. NVivo 12 Plus software was used to organize the data and manage the coding process, which included highlighting sections of text and linking them to relevant codes [38]. The coding unit was a meaningful segment of text, ranging from a phrase to several sentences that captured a relevant idea or experience. Codes were not treated as necessarily mutually exclusive, as one text segment could reflect multiple related ideas or experiences. When this occurred, the same text segment could be linked to more than one relevant code.

The analysis started with a thorough and iterative review of the transcripts. The codebook was constructed iteratively as coding progressed. Initial codes were developed from the early transcripts and then reviewed and refined through coding discussions. Across several rounds of coding, related codes were merged, overlapping codes were clarified, and code descriptions were revised to support consistent application across the dataset (S2 Appendix). Codes were grouped into categories that reflected identified themes based on their interrelationships [36,39]. This process formed the foundation for conceptualization, during which categories were examined to identify connections and uncover underlying concepts [39,40]. Following the Crafting Storyline approach, the themes were organized into four overarching concepts to reflect participants’ experiences [41]. Transition Theory was used as an interpretive framework to deepen the interpretation of the inductively developed findings. Concepts were then linked to Transition Theory to explore how they extended, supported, or challenged key elements of the theory [16,40].

To enhance the trustworthiness of the analysis, two researchers were involved in the analysis process [42]. Initial coding was conducted inductively, and the coding framework was reviewed and refined through discussion between the coders. Two researchers independently coded a subset of transcripts and compared coding outputs to assess consistency in how codes were defined and applied. Any disagreements were resolved through discussion until consensus was reached, and the codebook was refined accordingly. The codebook included the code name and a detailed description of each code. Code descriptions were used to guide coding decisions by clarifying what content fit within each code, distinguishing it from overlapping or unrelated content, and supporting consistent code application across the data.

Triangulation was supported in two ways. First, investigator triangulation was followed through the involvement of two researchers in coding and analysis. This allowed findings to be reviewed from more than one analytic perspective and supported discussion of similarities and differences in interpretation [42,43]. Second, data source triangulation was supported by the diversity of participants, who came from 15 countries and varied in gender, age, educational background, family status, time since arrival in Canada, and prior healthcare system experiences [43,44]. This variation allowed the analysis to compare patterns across different participant contexts and helped strengthen the credibility of the findings.

Trustworthiness was further supported through iterative coding discussions and review of study findings. A 1-2 page summary of the study findings was available to be shared with participants upon request. The Consolidated Criteria for Reporting Qualitative Research (COREQ) checklist was used to guide transparent reporting of the research team, study context, data collection, analysis, and interpretation [45]. Reflexive team discussions and the use of several direct quotations from different participants helped show how the findings were grounded in participants’ experiences and allowed readers to assess the connection between the data and the interpretations [36].

Following an approach adapted from Morton et al., participants were assigned pseudonyms indicating whether they were (Female [F] or Male [M]), participant number (e.g., P1), and the number of years they had been in Canada (e.g., 2yr). For example, FP1(<1yr) referred to a female participant who had been in Canada for less than 1 year.

### 2.6. Reflexivity

The study was conducted by female researchers with diverse professional backgrounds in pharmacy, medication management, and mental health. One member (YHA) was an international pharmacy graduate with lived experience transitioning to the Canadian healthcare system from another country. Two team members (YHA: BScPhm, MHSc; and AM: BSc, MSc) were graduate students with 2–3 years of experience in qualitative research at the time of the study. YHA conducted the interviews, while both YHA and AM carried out the data analysis. All study components were reviewed by the study supervisors (LD, LMM, and ADP) with expertise in pharmacy practice, medication management, care transitions, health equity, and social determinants of health. No prior relationship was established with participants before the study began.

### 2.7. Ethical considerations

Ethical approval for the study was granted by the University of Toronto research ethics board in Canada (REB# 44770). All participants provided informed written consent to participate in the study.

## 3. Results

### 3.1. Study sample

The study sample included 23 participants (8 male and 15 female) aged between 26-38 years old from 15 different countries with a diverse range of educational backgrounds. Participants varied in their length of time in Canada, ranging from less than one year to five years, which allowed the study to capture both early and later healthcare transition experiences. Participants also represented different regions and prior healthcare system contexts, including students who came directly from their countries of origin and others who had lived in another country or Canadian city before moving to Toronto.

Their social statuses varied including single living alone, single parent, as well as those who were married and/or living with a partner and/or children. Several participants had family responsibilities, including spouses, partners, pregnancy, and/or children, while others were living alone in Canada. This variation in country of origin, time since arrival, migration pathway, gender, age, educational background, and family composition helped capture a range of healthcare transition experiences among newcomer international students. No participant refused to answer or asked to skip any question, and none withdrew from the interview or at any stage of the study (*Refer to* Table 1 *for participants’ demographic data*).

**Table 1:** Participant demographic characteristics.

*(n=23)*		
**Countries of Origin**	***15 Countries***	
	India	3
	Egypt	2
	Bangladesh	2
	Kuwait	2
	Mexico	2
	Brazil	2
	Nigeria	2
	Kazakhstan	1
	Afghanistan	1
	Nepal	1
	Turkey	1
	Pakistan	1
	Qatar	1
	Hong Kong	1
	Guatemala	1
**Self-identified Gender**	Women	15
	Men	8
**Age Range**	25-30	6
	30-35	12
	35-40	5
**Social Status**	Married living with a partner, and children	12
	Married and living with a partner, no children	5
	Single living alone	3
	Single parent	2
	Married living with children	1

### 3.2. Concepts and themes

**Fig 2:**
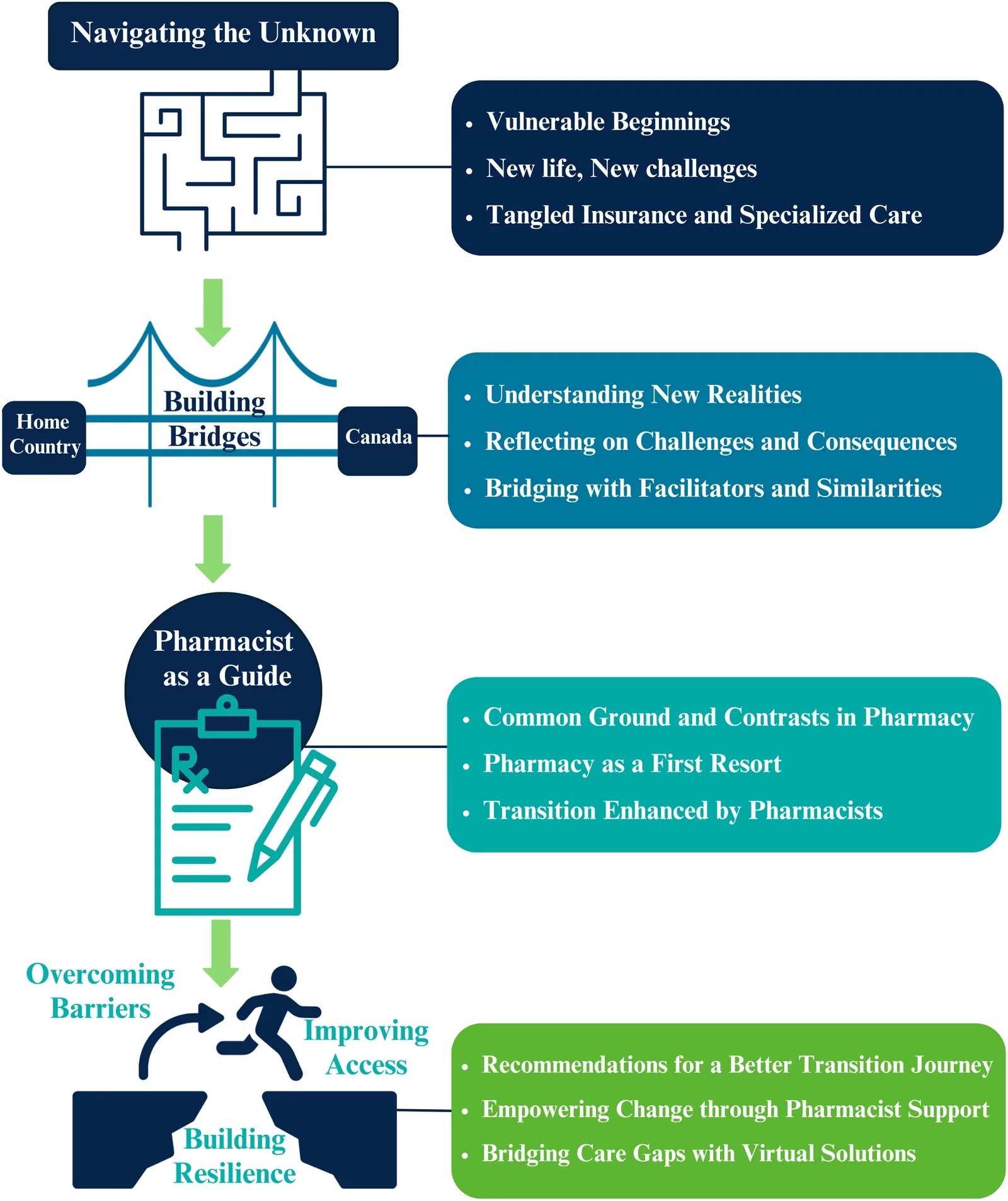
Conceptual model illustrating the key concepts and associated themes that reflect the experiences of newcomer international students when they transition to the Canadian healthcare system.

Following Crafting Storyline techniques from Golden-Biddle and Locke, the identified themes were categorized into four overarching concepts [41]. A conceptual model was developed by integrating these concepts to enhance understanding of participants’ transition to the Canadian healthcare system and the role of pharmacists in supporting them (Fig 2). By weaving these concepts into a cohesive storyline, the model captured the layered nature of participants’ experiences and emphasized the collective effort needed to support their transition [41].

#### 3.2.1. Concept 1: Navigating the unknown.

Within this concept, there were three main themes: vulnerable beginnings, new life new challenges, and tangled insurance and specialized care. Participants shared their initial experiences and impressions as they transitioned to a new healthcare system. Each participant's story was unique and influenced by their characteristics and needs which reflected the diverse challenges they encountered.

a. Vulnerable beginnings

In this theme, participants described the personal circumstances and vulnerabilities that surrounded their transition to Canada which reflected the complexity of adapting to life in a new country.

Several participants spoke about the emotional and social displacement they felt. The touching words of FP11(5yr) captured this: *“Of course, it felt really difficult at first. We left our jobs, families, parents, siblings, and homes, and came here to a totally different new life. So, I would say it was extremely difficult at first to get adjusted to everything.”* FP13(1yr) also shared how she navigated major life changes alone: “*I was recently married and I had to leave my husband back in my home country, and I was also pregnant at that time. It was hard to travel alone a long way, and I had just arrived in a new country with no one to help me.”*

Further complicating their transition, participants struggled to balance academic responsibilities, financial constraints, and family obligations. MP12(1yr) described: *“I was running between managing my studies and making sure that I get a good start in my PhD* *program, and then also managing the family responsibilities, especially finding a place to rent as a newcomer. I learned the hard way that it's not an easy task at all.”*

b. New life, new challenges

This theme highlighted the challenges and barriers participants faced during their initial navigation of the Canadian healthcare system due to differences from their home countries’ healthcare systems.

Several participants described challenges with the process of accessing care in Canada, particularly that a referral from a primary care provider is required to access specialist care. For example, FP1(<1yr) shared: *“Back home, it’s really easy to visit a specialist. You can go directly without a referral, but here, it’s more complicated because you have to see a family physician first, and then you may be referred to a specialist or not.”* Other participants noted that, compared to their home countries, accessing emergency services in Canada was perceived as more difficult due to longer wait times. Differences in access to mental health resources were also mentioned by FP18(1yr): *“I realized that a lot of things I took for granted back home are not so easy here, especially when it comes to mental health. I had six years of therapy, almost weekly or biweekly sessions [in her home country]. But here [in Canada], it feels like a struggle to access the same resources.”*

The lack of orientation on navigating the Canadian healthcare system was highlighted, particularly regarding what to do in emergency situations or when seeking urgent care. FP13(1yr) described her struggle: *“When I first came here, I was pregnant, and I didn't know where to go for checkups. It was my first pregnancy, and I had some bleeding. I didn’t know anything about the system here. I didn't know the condition of my baby at that time, and I wanted to know as soon as possible, how is my baby? But that took quite a long time, and it was very* *stressful.”* Moreover, FP4(<1yr) described initial confusion around basic healthcare terms, such as walk-in clinic, which in Canada refers to a medical clinic where patients can receive care without a prior appointment: *“I initially thought a walk-in clinic meant a clinic you could [physically] walk to because it is nearby and doesn’t require transportation.”*

c. Tangled insurance and specialized care

Participants described difficulties in understanding and accessing healthcare insurance, which were further complicated by having different insurance plans or pre-existing health conditions.

Citizens and permanent residents in Canada have access to physician and hospital services through government-funded insurance programs, for example, the Ontario Health Insurance Plan (OHIP). For international students, they may be covered under a patchwork of university insurance plans, for example, the University Health Insurance Plan (UHIP), which may vary in coverage from OHIP. Some participants shared challenges navigating the system, especially when family members were covered by different insurance plans. This mismatch in coverage made it difficult to access services, such as finding a family doctor who accepted both plans. One common scenario involved students being insured through UHIP, while their spouse or children were covered under OHIP. FP17(1yr) also reflected on the differences in insurance coverage between both countries: *“In my country, my employment insurance covers everything 100%. I don’t even pay for extra services like physiotherapy, dental, eye exams, or even glasses. But here, I’m not sure how I can afford all that.”*

Participants with chronic conditions and other health-related needs faced additional barriers. MP2(3yr), who lives with diabetes, hypertension, and high cholesterol, shared: *“When I moved to Canada, I didn’t understand how insurance coverage works. It's unclear whether my* *medication will be covered or if I'll need to pay out of pocket.”* FP18(1yr) described the added challenges of receiving an ADHD (Attention Deficit Hyperactivity Disorder) diagnosis: *“I was diagnosed with ADHD, and I felt really sad, not just because of the diagnosis, but because it meant I needed more support, like a psychiatrist and therapy. I knew it would be hard to access these services and might not be covered.”*

#### 3.2.2. Concept 2: Building Bridges.

This concept included three main themes: understanding new realities, reflecting on challenges and consequences, and bridging with facilitators and similarities. Together, these themes captured how participants took initial steps toward adaptation and began building knowledge to bridge healthcare gaps between the healthcare systems in their home countries and in Canada.

a. Understanding new realities

This theme described how participants used self-education and community resources to understand the new healthcare system; however, this sometimes led to misinformation and delayed care.

The internet served as a key resource for several participants who used it to search for information about the healthcare system, such as available services, insurance coverage, and eligibility. For other participants, support from their social networks played a key role, MP15(1yr) noted: *“We knew about the healthcare system here by asking our friends, neighbours, other students, and colleagues.”* In anticipation of potential challenges accessing medications in Canada, all participants reported bringing medication supplies from their home countries based on what they had heard or read.

While self-education and community advice were essential for adapting to the new healthcare system, they sometimes led to misconceptions that made the adjustment process more difficult. For example, some reflected cultural expectations as MP12(1yr) shared: *“In our community, we often wonder why we aren't given more medication to be cured quickly and better.”* Fear stemming from misinformation also caused delays in seeking care. FP13(1yr): *“I heard there would be restrictions, like if you're pregnant, you can’t continue as a student. I wasn’t sure if I could bring my pregnancy medications. I was really afraid, what if they found out and deported me? So, I didn’t bring them. I even thought my prescription from back home would work here, but it didn’t.”* Moreover, FP21(1yr) expressed uncertainty about her healthcare rights as an international student: *“I thought I could not have my own family doctor, and I could only go to the university's health center to see a doctor.”*

b. Reflecting on challenges and consequences

In this theme, participants reflected on their experiences, seeking to understand and explain the challenges they faced from their perspective and how these challenges affected their lives.

Several participants pointed to broader systemic issues as explanations for the difficulties they encountered. One commonly discussed issue was the lack of clear and accessible healthcare information. A shortage of healthcare professionals was also identified as a major contributing factor, as FP8(2yr) mentioned: *“I feel that in Canada, they have labour shortages in terms of doctors, nurses, technicians, and pharmacists. So, the wait time is way too long.”* The medical licensure process for foreign-trained healthcare professionals was viewed as a possible contributor to this shortage. In addition, the centralized role of physicians was criticized for contributing to delays. FP18(1yr) noted: *“Everything seems to go through a physician. This is one of the reasons why it's so hard to get an appointment with one.”*

Participants also described how these systemic challenges affected their well-being and academic life. Several participants described how delays in access to care and overburdened services led to misdiagnoses, medical errors, and increased stress. MP2(3yr) shared how these barriers had significant emotional and familial impacts: *“My son had speech delay and behavioral issues, but the waiting list to get help here was so long. This made me consider giving up my PhD, but I decided to send him back home for a while to see if this might help with his development. This experience has been very hard on us as a family.”* Avoidance also became a coping strategy for FP1(<1yr), who mentioned: *“Because of these difficulties in getting an appointment and all the delays, I often ignore small health problems, but I’m still afraid they could develop into something more serious.”*

c. Bridging with facilitators and similarities

This theme highlighted facilitators and similarities between the healthcare systems in Canada and participants’ home countries which helped ease their transition and access to care.

University organizations served as a key source of guidance that helped some participants navigate the system and connect with primary care services. Financial assistance, including access to publicly funded programs, further eased the transition, as FP20(1yr) shared: *“My kid was able to get into the free Healthy Smiles program, and she received dental treatment at no charge.”* Supportive healthcare professionals also played an important role, especially when they demonstrated understanding and flexibility around common issues such as insurance coverage.

Some participants identified similarities between the healthcare systems in Canada and their home countries, as FP7(4yr) noted: *“It was just as affordable here as it was back home. If you were working or studying, you got insurance, and you don’t really pay much for medical services.”* Interestingly, FP9(<1yr) highlighted that differences between healthcare systems could also serve as a facilitator: *“Something that's different between my country and here is that back home, citizens are much more privileged compared to residents. So, if a resident and a citizen approach a healthcare facility at the same time, the citizen is much more likely, probably 99%, to see a doctor first and get medical care first, not like here, where everyone is treated the same.”*

#### 3.2.3. Concept 3: Pharmacist as a guide.

Three key themes were included in this concept: common ground and contrasts in pharmacy, pharmacy as a first resort, and transition enhanced by pharmacists. These themes reflected participants’ experiences with using pharmacy services and described the support they received from pharmacists during their transition to the Canadian healthcare system.

a. Common ground and contrasts in pharmacy

This theme examined both the similarities and differences in pharmacy practice between Canada and participants’ home countries to explore what helped or hindered their adjustment to the new healthcare system.

Several participants noted that familiar aspects of pharmacy practice helped them understand what to expect from pharmacists in Canada and made pharmacy services feel more approachable and accessible, including shared processes such as medication dispensing and counselling. MP19(1yr) mentioned: *“I don’t think there’s much difference from my home country which made it easier for me to use their [Canadian pharmacies’] services.”*

At the same time, other participants highlighted structural differences. Several noted that pharmacists in their home countries played a broader role. FP1(<1yr) described how this difference affected her willingness to use pharmacy services: *“In my country, I could ask the pharmacist about many health issues, and they would give me the best medication without a* *prescription. Here, the pharmacist’s role feels more limited, so I'm hesitant to go there [community pharmacy in Canada] because I'm not sure if it will be helpful.”* Some participants described other challenging differences including stricter prescription requirements, longer wait times, and higher medication costs. MP2(3yr) explained: *“UHIP doesn’t cover medications, and without extra insurance, prices can be really high compared to my home country. That makes it hard for us to get the medications we need.”*

b. Pharmacy as a first resort

This theme reflected participants’ perspectives on community pharmacies as their initial point of contact upon arriving in Canada and how this helped them adapt to an unfamiliar healthcare system.

Several participants viewed community pharmacies as an accessible resource for support and guidance, as FP20(1yr) described: *“I think the pharmacy is a great entry point to the healthcare system here. Pharmacists can direct patients to whatever is appropriate to do. You don't really need to have insurance to talk to a pharmacist*.” The benefits of the widespread availability of pharmacies were also highlighted by FP9(<1yr): *“There is a pharmacy everywhere in Canada. So, if you are new, it is easy to go to the pharmacy and ask about any information you need about the healthcare system here.”* Moreover, some described positive experiences during their initial contact with the healthcare system where pharmacists who spoke their native language helped overcome communication barriers and enabled them to receive the care they needed.

Another factor that added to the convenience of pharmacies for participants as newcomers was their experiences with using the e-Prescribing System, where prescriptions are sent electronically from clinics and hospitals to a nearby pharmacy chosen by the patient [46]. For example, FP13(1yr) shared: *“The prescription pickup system here is great. It’s really convenient because we don’t have to go far to get our medications. It saves us time in our busy schedules, especially when we were still getting used to the transportation here.”* Others highlighted the accessibility of other essential services, such as receiving COVID-19 and influenza vaccines at community pharmacies.

c. Transition enhanced by pharmacists

This theme highlighted how pharmacists played an ongoing role in supporting participants as they began engaging more actively with the healthcare system and began navigating different services.

Several participants shared how pharmacists increased their confidence in using unfamiliar medications, as FP18(1yr) noted: *“Every time that I get medication here, the pharmacist gives me instructions about my medication, such as how many times I should take it and explains the side effects I might experience and what to do if that happens. This makes me feel safe, especially with the new medications I’m not used to.”* Pharmacists were also recognized for providing timely care for minor health concerns, FP8(2yr) described: *“During the weekend, my daughter was vomiting, and the pharmacist suggested using some over-the-counter drugs. My daughter's stomach flu settled in just one day.”*

Beyond medication-related support, pharmacists assisted with a range of other healthcare needs, MP10(4yr) shared: *“When I was getting a brace for my ankle, the pharmacist was very helpful. He showed me how to put it on and explained what to be careful about.”* FP21(1yr) was referred by the pharmacists to see a physician for a post-COVID cough that required a prescription inhaler. After obtaining it, the pharmacist continued to provide guidance as she shared: *“I had never used an inhaler before. Luckily, the pharmacist explained how to use it and* *recommended watching some YouTube videos for extra guidance.”* Another participant also described how pharmacists offered helpful guidance on vaccination schedules, including flu shots and COVID-19 booster doses, supporting both individual and family health.

#### 3.2.4. Concept 4: Overcoming barriers, building resilience, and improving access.

This concept included strategies suggested by participants to overcome identified barriers and improve the healthcare transition for newcomer international students and their families including the educational role of pharmacists and the transformative potential of adopting virtual care. Three key themes were identified: recommendations for a better transition journey, empowering change through pharmacist support, and bridging care gaps with virtual solutions.

a. Recommendations for a better transition journey

This theme explored participants’ proactive contributions, including recommendations to address healthcare gaps and improve the accessibility and relevance of services to better meet their needs.

Several participants emphasized the importance of improving education and awareness for newcomer international students, especially upon arrival, as FP8(2yr) stated: *“I think the first thing that needs to be done is raising awareness. People come here as international students, and they have no idea how the healthcare system works.”* They also called for more structured educational materials and targeted outreach strategies to help students understand how to access and navigate healthcare services. Other participants also highlighted the university's important role in educating newcomer international students about the healthcare system. FP22(1yr) suggested: *“Maybe part of the university package could be an information sheet that includes* *examples of clinics that accept UHIP and are open to new international students. This way, we can access healthcare immediately after we arrive.”*

In addition to formal resources, other participants highlighted the value of peer support and social connection in easing the transition. They proposed creating opportunities for newcomer international students with more experience to support newcomers by answering questions and sharing tips about the healthcare system. MP10(4yr) suggested: *“Preparing some guides and organizing social events could be really helpful because people will feel more comfortable talking and exchanging experiences.”*

b. Empowering change through pharmacist support

Participants in this theme shared insights on improving medication access and expanding the scope of pharmacy practice to better address their complex healthcare needs and ultimately improve health outcomes.

Several participants endorsed the idea of leveraging community pharmacists as key educators when asked how pharmacists can better support their needs. Suggestions included creating a list of pharmacies with available pharmacists to assist newcomer international students, offering targeted educational sessions about the healthcare system, and promoting these services, especially within university residences, to increase awareness. To address privacy concerns, FP11(5yr) recommended: *“Having a dedicated private space at the pharmacy would make us feel more comfortable discussing our needs.”* FP21(1yr) also emphasized the importance of addressing language barriers: *“It would be very helpful to know which pharmacists can speak more than one language. This will help us and our families more.”*

Some participants highlighted the need for clear information about the scope of practice for different healthcare professionals to understand which services to seek when they have health concerns. They stressed the importance of proactive resources about medication equivalents and availability. FP7(4yr) explained*: “I really need to know what kind of medications are available over the counter and what are available through prescription.”* In addition, several participants advocated for expanding pharmacists’ roles to improve accessibility. FP8(2yr) also proposed structural changes to reduce pressure on the healthcare system: *“Maybe a good idea is relaxing the licensure process for foreign-trained pharmacists to ensure good access to the pharmacy for everyone.”*

c. Bridging care gaps with virtual solutions

This theme included participants’ suggestions on how pharmacists could offer virtual care services to further support them, highlighting potential benefits and the optimal timing for delivering these services.

Several participants emphasized the educational potential of virtual care services and suggested innovative ways to leverage community pharmacists’ expertise. FP4(<1yr) mentioned: *“I think pharmacists can provide some sort of educational Zoom meetings or prepare online videos and posters to make students aware of the healthcare system here and how they can use the available services.”* The use of a standard and user-friendly virtual care platform was also recommended.

Moreover, participants discussed the benefits of virtual care. For example, FP13(1yr) highlighted its convenience: *“We can learn about the healthcare system here and how to access many services using virtual care. We can access this easily from anywhere at any time.”* Additional benefits included saving time, enhancing accessibility, reducing barriers, and improving efficiency. FP20(1yr) also stated*: “Virtual care makes it easier to offer multilingual* *services for international students. It takes time for us to adapt to the language and communicate our health information accurately in English.”*

Participants had varied perspectives on the optimal timing for virtual care services. For example, FP18(1yr) mentioned: *“We need a virtual source of information before coming because I'm always very worried, and I did all these things that I could have simply avoided, like bringing medication that I might not need or could easily get in Canada.”* Meanwhile, FP23(1yr) emphasized a more flexible approach: *“I think these virtual services should be available all the time because everyone is different and has different priorities. Some people like to plan ahead, and others have so much going on before they move.”*

## 4. Discussion

International students represent a large and essential segment of Canada’s population [10,11]. As newcomers, they encounter multiple layers of adjustment including academic, cultural, and healthcare-related challenges [13,14]. Despite Canada’s commitment to equitable healthcare, systemic gaps continue to limit access for vulnerable groups such as newcomer international students. This study contributes to a deeper understanding of these students’ lived experiences with healthcare transition and explores how pharmacists, as accessible healthcare providers, can help bridge some of these care gaps. It also offers novel insights such as the health risks that may arise when students lack information about the new healthcare system including safe medication practices, as well as the mixed influence of informal supports that can potentially guide or misguide them during early transition. In addition, it highlights several sources of mental and emotional burden related to difficulties accessing care while emphasizing the promising role of virtual care in reducing uncertainty, addressing misinformation, and supporting students before and after their arrival in Canada.

The participants’ unique stories were woven together to form a coherent conceptual model. They described their initial uncertainties and emotional journeys as they encountered various challenges and barriers while adapting to their new life in Canada *(Concept 1: Navigating the Unknown)*. Efforts to bridge the gap between their previous healthcare experiences and the Canadian system involved assessing their situation and seeking support from various sources as a step forward to integrate into the new system *(Concept 2: Building Bridges).* Participants expressed support for pharmacists, who were largely viewed as accessible and knowledgeable allies amidst the complexities of the new healthcare system *(Concept 3: Pharmacist as a Guide).* Importantly, participants proposed several recommendations to address the challenges they struggled with and improve access to healthcare services which highlighted their resilience and their active role in facilitating healthcare transition for themselves and future newcomer international students *(Concept 4: Overcoming Barriers, Building Resilience, and Improving Access)* (Fig 2).

Exploring these findings through the lens of Transition Theory was well-suited for this study because it reinforced the multifaceted nature of the adaptation process [16,17]. The findings of this study reflected how newcomer international students often navigated multiple, overlapping transitions. Drawing on Meleis et al.’s description of transition patterns, Fig 1 situates these findings within Transition Theory by illustrating how participants’ healthcare transition was shaped by such intersecting experiences. These included the situational transition of relocating to a new country, the developmental transition of becoming more independently responsible for themselves and their families, and the health-illness transition of managing new or ongoing health conditions within a different healthcare system. Additionally, they experienced an organizational transition as they adapted to the structure, policies, and processes of the Canadian healthcare system, such as navigating insurance coverage, referrals, and scopes of practice (Fig 1). These interconnected transitions highlighted the importance of holistic and system-informed supports to ease adaptation and promote better healthcare outcomes.

The four overarching concepts identified in the analysis aligned with key components of Transition Theory, including types and patterns of transitions, transition conditions, process indicators, and outcome indicators [16,17]. Concept 1 “Navigating the Unknown”, reflected types and patterns of transitions, which in Transition Theory refer to the kinds of changes individuals experience and whether transitions occur as single, multiple or sequential [16,17]. Participants described the overlapping nature of their transition as they encountered healthcare-related changes while adjusting to life in Canada. This concept also reflected inhibiting transition conditions, described in Transition Theory as factors that can hinder a healthy transition [16,17]. Inhibiting conditions included limited knowledge of the Canadian healthcare system, uncertainty about where to seek care, confusion around insurance and referral pathways, emotional stress, language and communication challenges, and difficulty accessing timely care.

Concept 2 “Building Bridges”, reflected transition conditions and process indicators. Misinformation, limited orientation, and uncertainty about healthcare rights acted as inhibiting transition conditions while previous healthcare experiences, peer and community advice, university supports, and similarities between healthcare systems acted as facilitating transition conditions. This concept also reflected process indicators, a component of Transition Theory that captures how individuals engage with and respond to transition over time [16,17]. Participants’ online searching, self-education, comparisons with healthcare systems in their countries of origin, and reliance on community knowledge showed how they tried to make sense of the new healthcare environment and identify where to seek care.

Concept 3 “Pharmacist as a Guide”, reflected facilitating transition conditions within the healthcare transition process. Pharmacist accessibility, medication counselling, and guidance helped participants understand medication-related needs, clarify pharmacy and insurance-related questions, and identify when or where to seek further care. This concept showed how pharmacists could support participants’ confidence, coping, and connection to care, particularly when participants faced uncertainty about professional roles, medication access, or appropriate points of care.

Concept 4 “Overcoming Barriers, Building Resilience, and Improving Access”, reflected outcome indicators, shown in Transition Theory as signs of progress toward a healthy transition, such as more confidence, coping, and ability to manage new roles or situations [16,17]. Participants’ recommendations for structured education, pharmacist-led guidance, pre-arrival support, and virtual care reflected movement from uncertainty toward greater awareness and identification of needed support. The recommendations also showed participants’ active role in identifying strategies that could improve healthcare transition for future newcomer international students.

Therefore, the findings support and extend the application of Transition Theory by showing how healthcare transition for newcomer international students occurs across institutional, medication-related, insurance-related, digital, and family contexts. These findings add contextual specificity to the application of the theory by illustrating how transition conditions may be experienced through practical healthcare-navigation tasks, including understanding medication access, insurance coverage, professional roles, referral pathways, virtual resources, and family-related healthcare responsibilities.

Most participants described confusion and limited knowledge about the Canadian healthcare system as they transitioned to their new life, including how to access different healthcare services and navigate the referral process. These findings are consistent with those reported in previous studies which identified unfamiliarity with the healthcare system and lack of general healthcare information as one of the most significant barriers to healthcare access for international students [47,48]. However, this study also shed light on the potential serious health risks that can arise from this lack of information including risks to pregnant individuals and their babies, as well as consequences for those living with chronic conditions. It further showed that some students ignored early health concerns because of difficulties accessing care, even while worrying that these issues could progress into something more severe.

This study also identified several barriers to effectively using healthcare services such as communication and language difficulties, long wait times, challenges in understanding health service information, and confusion about the roles and responsibilities of different healthcare professionals. In other countries, such as Australia and the United States, similar challenges were common among newcomer international students, and other studies have found that these were often so overwhelming that some chose to avoid university health support services altogether and instead attempted to manage their health issues independently [49,50]. Furthermore, bringing medications from their home countries to overcome differences in healthcare systems was reported by all study participants and has also been previously identified as a common practice among international students in Canada and other countries [47,49]. This study helped explain why this common practice might occur by identifying factors such as limited health-insurance coverage for medications, high medication costs, and uncertainty about which medications require a prescription in Canada. In addition, the medications students brought from their home countries were often not reviewed for appropriateness, safety, correct use, or provided with guidance on storage or disposal if not used. These findings highlight an overlooked safety and environmental gap and emphasize the need for targeted educational interventions for newcomer international students.

Moreover, this study revealed the complexities newcomer international students faced in understanding and accessing the university health insurance plan. These issues affected how students navigated the Canadian healthcare system and made decisions about their care. Similar limitations and the resulting financial burden have been reported in previous studies [47,51]. Such challenges could have influenced students’ decisions to fill prescriptions, forced some to purchase costly private health plans, and led others to delay or cancel dental visits due to financial constraints [47,48].

Mental health was another recurring concern raised by participants who described experiencing anxiety and emotional stress. These challenges were often exacerbated by academic pressure, social isolation, and limited information on how to access mental health services. Participants also described several sources of emotional strain such as difficulty accessing the mental health support they previously relied on, delays in receiving essential services for themselves or their children, and uncertainty about how to navigate urgent healthcare needs, especially during pregnancy or other vulnerable periods. These findings aligned with previous research showing elevated rates of psychological distress, including depression and anxiety among international students in Canada and globally [8,50,52]. In addition, evidence from the literature suggested that a structured health education intervention significantly improved international students’ confidence in seeking mental health support and led to significant reductions in reported mental health issues [53].

Several participants reported that self-education, advice from peers, and certain similarities between the Canadian healthcare system and those in their countries of origin helped facilitate their transition. The study also highlighted the dual effect of the support students relied on. While self-education and community advice helped them begin navigating the healthcare system, these same strategies sometimes contributed to misconceptions that complicated their adjustment, offering a novel insight into the mixed role these supports play during early healthcare transition.

Pharmacists were viewed as accessible and trusted allies who supported healthcare transition by enhancing participants’ understanding of medication and providing timely guidance and care. Participants’ experiences showed that understanding the broader context of their challenges was essential for interpreting their perspectives on the pharmacist's role. These experiences reinforced that pharmacists represent an important component of the broader support needed to help newcomer international students achieve better healthcare transition and improved care experiences. Therefore, participants advocated for enhancing the pharmacist’s role to better meet their healthcare needs. This was also supported by other studies suggesting that expanding pharmacists’ scope of practice and ensuring appropriate remuneration serve as an important step toward reinforcing primary care and building a stronger healthcare system [54,55]. To further support their role as key educators, participants also suggested that pharmacists could deliver structured sessions on navigating the Canadian healthcare system and emphasized the importance of promoting these services to raise awareness. To address language barriers, participants suggested that the university share information about pharmacists available to help and whether they speak additional languages.

While this study mainly explored the pharmacist’s role, the findings should be interpreted within the broader healthcare system context. Participants’ experiences showed that their needs require support from different healthcare providers and services, including family physicians, specialists, nurses, mental health services, dental care, university health services, and insurance or administrative guidance. Therefore, pharmacists should be viewed as complementary to, rather than a replacement for other healthcare providers. At the same time, examining pharmacists’ role in this context is important because newcomer international students may face delays or uncertainty when accessing care, particularly when they are unfamiliar with the roles of different healthcare providers, referral pathways, or which service is appropriate for their concern. In this context, pharmacists could serve as an accessible early point of contact to provide medication-related guidance, clarify pharmacy and insurance-related questions, identify when medical assessment is needed, and direct students to appropriate healthcare services. This aligns with evidence that community pharmacies are geographically accessible and that pharmacists’ roles in Canada have expanded beyond dispensing to include medication management, patient education, referral, and collaboration within primary care [56,57].

This focus was also relevant because many of the challenges participants described aligned with the scope of pharmacy practice in Canada, including addressing uncertainty about prescription requirements, over-the-counter medications, medication coverage, medications brought from home, and where to seek timely advice. Integrating pharmacist-led education or navigation support into broader university orientation and healthcare access initiatives could help address several reported barriers faced by newcomer students, promote safer medication use, reduce misinformation, improve referral to appropriate services, and support earlier connection to appropriate care. This system-level approach is consistent with broader calls to make healthcare more accessible, culturally responsive, and better aligned with newcomer needs [7].Additionally, participants proposed broader improvements to the current support system. These included developing comprehensive and standardized resources, such as a university information package on how to access and navigate healthcare services. Virtual care was highlighted as a potential flexible and convenient way to receive this information, especially for students with busy schedules or limited access to in-person services. This is supported by previous research showing that virtual care is a feasible and effective approach to promote healthy behaviors, while also improving knowledge, medication adherence, treatment outcomes, and communication with pharmacists [58–60]. Furthermore, the study provides important insights into how virtual care could serve as a bridge across international boundaries, support both early and ongoing transition needs, and help reach more students. The findings show that virtual care may proactively assist students with their healthcare needs and prepare them for the Canadian healthcare system before and after arrival. Participants explained that pre-arrival virtual support could reduce uncertainty, address misinformation, and provide timely guidance on accessing medications and navigating the healthcare system.

As this qualitative study was conducted at one academic institution, our aim was to gain a comprehensive understanding of the specific healthcare transition experiences, challenges, and needs described by the participants in this study. Such insights reflect the perspectives of these individuals within their unique institutional and healthcare contexts. While the findings offer valuable and detailed perspectives, they are intended to provide depth of understanding rather than broad generalizability. Participants’ lived experiences revealed substantial barriers, unmet needs, and institutional gaps that require attention. A key finding of this study is that it supports critiques of the policy narrative that frames international students as “ideal immigrants” who can easily integrate into Canadian systems [61]. Despite their meaningful contributions to academic and cultural life, international students are often overlooked and under-supported, especially when it comes to navigating foundational services such as healthcare [48,49,61]. Although there has been a considerable number of studies on the challenges faced by refugees and other immigrant populations, there remains a dearth of literature focused on addressing the specific needs of international students, despite their vulnerability to experiencing healthcare access barriers [48,61].

## 5. Limitations

While this study provides valuable insights into the healthcare transition experiences of newcomer international students in Canada, several limitations should be acknowledged. The study focused on students enrolled at a single academic institution (U of T), which may limit the transferability of findings to students in other educational institutions or regions. U of T has specific institutional resources, international student supports, health-related services and resources, and community partnerships that may differ from those available at other universities or colleges. In addition, participants were studying in Ontario, where healthcare coverage, insurance arrangements, pharmacy services, and access pathways may differ from those in other provinces or countries. Differences in institutional support structures, local healthcare access, and community services could lead to different experiences. Moreover, although the sample included participants from 15 different countries and reflected a range of social and educational backgrounds, the study may not fully capture the breadth of diversity within the international student population in Canada, which includes individuals from over 200 countries.

## 6. Future directions

This study highlights the need for further research to expand the understanding of healthcare transition among newcomer international students and their families to identify scalable solutions that address their access barriers. Future research should include students from multiple institutions across diverse geographic and institutional settings to capture a wider range of experiences. Additionally, longitudinal studies would be particularly valuable in understanding how newcomer international students’ healthcare knowledge, access, and confidence change over time as they adapt to the Canadian healthcare system. Evaluating pharmacist-led or university-based interventions including virtual care models, may also help determine best practices for supporting this population in a sustainable and culturally responsive way.

## 7. Conclusion

Newcomer international students in Canada face complex challenges when navigating the healthcare system, including unfamiliarity with services, differences in insurance coverage, and mental health concerns. Their experiences highlight key barriers but also reveal opportunities for pharmacists to play a stronger role in supporting healthcare transition. Expanding pharmacy-led education, improving system navigation, and offering virtual care may improve access and health outcomes. These findings point to the need for culturally responsive, system-level strategies that address the healthcare needs of this underserved population.

## Supporting information

S1 Appendix Updated interview guide. (PDF)

S2 Appendix Coding book and analytic mapping of codes, themes, and concepts. (PDF)

S3 Appendix Consolidated criteria for reporting qualitative studies (COREQ): 32-item checklist. (PDF)
