# Supplementary material for: A qualitative study exploring healthcare transition experiences among newcomer international students in Canada and opportunities for pharmacist support
Source: PLoS One. 2026 Aug 28;21(8):e0356780. doi: 10.1371/journal.pone.0356780 (PMC13524333; doi:10.1371/journal.pone.0356780)
Supplement: Supplementary file 1 [file pone.0356780.s001.pdf]

S1 Appendix: Updated* Interview guide

*(Refined/Added questions are underlined)

The interview guide (Interview duration is approximately 30-60 minutes)

Demographic data:

- Name:
- Age:
- Gender:
- Date of arrival to Canada:
- Living alone or with family/partner:

List of questions:

1. **Could you please tell me about yourself and your program of study?**

   Probes:

   – What led you to choose your current program of study?

   – Tell me more about your life here, have you met any new people or made new friends?

   – How did it feel to leave your country of origin and move to Canada?

2. **I'm mainly interested in transitioning to the Canadian healthcare system by international students and their families, could you please tell me more about your experience?**

   Probes:

   – Where did you get the information about the Canadian healthcare system during your initial contact?

   – What are the main similarities and differences between the healthcare system here and in your country of origin?

   – What are the main challenges you faced during your initial contact with the Canadian healthcare system? And why do you think these challenges happened?

   – Could you please tell me about the impact of such challenges on your everyday life?

3. **Could you please tell me about your experience with medication changes/ disease management when you moved to Canada? And the main barriers you faced? What went well?**

   Probes:

   – Do you or any member of your family have a chronic disease or regularly use specific medications, vitamins, food supplements, or herbal products? If yes, could you please tell me more about it?

- Some students mentioned bringing medications from their home country, are you familiar with this?
- Have you used any pharmacy services or healthcare services to help you with your medications? If yes, how did you find these services?
- Are you aware of the minor ailments that pharmacists are allowed to prescribe medications for?
- In your opinion, what are the main differences between the pharmacist's role here versus their role in your home country?
- What do you think of the pharmacists' role in Canada?

4. **What actions would you consider helpful in supporting international students' transition to the healthcare system here in Canada?**

   Probes:
   - How can the pharmacist help international students and their families with transitioning to the Canadian healthcare system? more specifically in addressing any medication-related issues?
   - To better support international students and their families, what can be done to improve pharmacy care services?

5. **Could you please tell me about your experience with using virtual care[1]?**

   Probes: if they have experience with using virtual care:
   - What are the pros and cons of using in-person versus virtual services?
   - What do you think of using virtual care to deliver services to help international students and their families transition to the Canadian healthcare system?
   - What is the optimal time to receive the support services for transition to the healthcare system?
   - What are your suggestions to improve virtual care services?

[1] According to the Canadian Institute for Health Information, virtual care has been defined as "any interaction between patients and/or members of their circle of care, occurring remotely, using any forms of communication or information technologies, with the aim of facilitating or maximizing the quality and effectiveness of patient care".
