# Supplementary material for: A qualitative study exploring healthcare transition experiences among newcomer international students in Canada and opportunities for pharmacist support
Source: PLoS One. 2026 Aug 28;21(8):e0356780. doi: 10.1371/journal.pone.0356780 (PMC13524333; doi:10.1371/journal.pone.0356780)
Supplement: Supplementary file 2 [file pone.0356780.s002.pdf]

S2 Appendix

Coding Book

Number	Code Name	Description	Example Quote
1	First experiences/impressions	Describing the initial experiences/impressions of international students and their families as they transitioned to the Canadian healthcare system.	*"The first encounter I had was when my daughter had an emergency. She had swelling in her eye, and it was very acute. We didn't know how to start the process or how to approach it."* FP7(4yr)
2	Transition vulnerabilities/needs	Describing the vulnerable status of newcomer international students and their families during their transition to the Canadian healthcare system.	*"Of course, it felt really difficult at first. We left our jobs, families, parents, siblings, and homes, and came here to a totally different new life. So, I would say it was extremely difficult at first to get adjusted to everything."* FP11(5yr)
3	Students personal characteristics	Describing students' specific characteristics related to their backgrounds, socioeconomic status, education, and accessibility factors, either in their home countries or in Canada.	*"One good thing is that we have a lot of doctors in our family. … I'm not a medical doctor, but I work in the health and nutrition sector, so I do know a little bit about this, even though I'm not clinical. We have medical people, so we do get guidance from them and make sure that we are not overdoing anything, like any overdose or things like that."* MP12(1yr)
4	Students/families medical history	Describing the medical history of international students and their families, including chronic diseases, use of medications, herbal products, or vitamins.	*"When I moved to Canada, I didn't understand how insurance coverage works. It's unclear whether my medication [for diabetes] will be covered or if I'll need to pay out of pocket."* MP2(3yr)
5	Challenges/barriers to learn about/access/use Canadian healthcare services	The barriers and challenges faced by international students and their families in using Canadian healthcare services, whether personal, related to accessibility, or due to healthcare provider-related issues.	*"For a specific health problem, even basic issues, I didn't know where to find the right doctor or which pharmacy I should go to. When I went to the medical center, I didn't know which doctor they were going to assign me to. It felt random, like whoever was present there would see me."* MP15(1yr)

6	Differences between both healthcare systems	Describing the main differences between the overall healthcare systems in students' home countries and the Canadian healthcare system.	*"I realized that a lot of things I took for granted back home are not so easy here, especially when it comes to mental health. I had six years of therapy, almost weekly or biweekly sessions [in her home country]. But here, it feels like a struggle to access the same resources."* FP18(1yr)
7	Potential/unverified misconceptions of the Canadian healthcare system/medical misinformation	Including the misinformation international students have/had about healthcare services or healthcare providers.	*"I thought I could not have my own family doctor, and I could only go to the university's health center to see a doctor."* FP21(1yr)
8	Self-educating/support	Describing how international students learned about or overcame barriers to accessing healthcare services on their own.	*"I learned about the healthcare system by Googling it and looking for information online about what medications to use for common symptoms, such as colds or fever."* FP17(1yr)
9	Family/friends support/shared knowledge/recommendations	Includes the recommendations or information provided by international students' friends, colleagues, or neighbours on how to navigate/access the healthcare system in Canada.	*"We knew about the healthcare system here by asking our friends, neighbours, other students, and colleagues."* MP15(1yr)
10	Self-reasoning/explanations for challenges	Includes the explanations provided by international students for the challenges they faced during their transition to the Canadian healthcare system.	*"Everything seems to go through a physician. This is one of the reasons why it's so hard to get an appointment with one."* FP18(1yr)
11	Impact of challenges on everyday life/study/health	The impact that international students experienced on their everyday lives/study/health due to the challenges they faced following their transition to the Canadian healthcare system.	*"Because of these difficulties in getting an appointment and all the delays, I often ignore small health problems, but I'm still afraid they could develop into something more serious."* FP1(<1yr)
12	Similarities between both healthcare systems	Describing the main similarities between the overall healthcare system in students' home countries and the Canadian healthcare system	*"It was just as affordable here as it was back home. If you were working or studying, you got insurance, and you don't really pay much for medical services."* FP7(4yr)
13	Facilitators to learn about/access/use the Canadian healthcare services	The facilitators that enabled international students to access Canadian healthcare services including	*"My kid was able to get into the free Healthy Smiles program, and she received dental treatment at no charge."* FP20(1yr)

		personal, accessibility, and healthcare provider-related factors.	
14	Similarities between both pharmacy systems	Describing the main similarities between the pharmacy systems in students' home countries and in Canada. The pharmacy system refers specifically to the structure, roles, and services provided within pharmacy practice, such as medication dispensing, pharmacist-patient interactions, and health education.	*I don't think there's much difference from my home country which made it easier for me to use their [Canadian pharmacies'] services."* MP19(1yr)
15	Differences between pharmacy systems in both countries	Describing the key differences between the pharmacy systems in students' home countries and in Canada. This includes variations in pharmacists' roles, scope of practice, medication dispensing policies, access to medications, and the types of services provided specifically within pharmacies.	*"In my country, I could ask the pharmacist about many health issues, and they would give me the best medication without a prescription. Here, the pharmacist's role feels more limited, so I'm hesitant to go there [community pharmacy in Canada] because I'm not sure if it will be helpful."* FP1(<1yr)
16	Pharmacy services	Includes the pharmacy services that international students use to help them with their healthcare needs.	*"Every time that I get medication here, the pharmacist gives me instructions about my medication, such as how many times I should take it and explains the side effects I might experience and what to do if that happens. This makes me feel safe, especially with the new medications I'm not used to."* FP18(1yr)
17	Pharmacy services satisfaction level	Describing the extent to which students are satisfied or dissatisfied with the pharmacy services provided to them.	*"When I was getting a brace for my ankle, the pharmacist was very helpful. He showed me how to put it on and explained what to be careful about."* MP10(4yr)
18	Factors impacting learning about/ accessing/using pharmacy services in Canada	Includes both barriers and facilitators that impact international students' ability to use pharmacy services in Canada, whether personal, accessibility-related, or pharmacist-related factors.	*"There is a pharmacy everywhere in Canada. So, if you are new, it is easy to go to the pharmacy and ask about any information you need about the healthcare system here."* FP9(<1yr)

19	Pharmacist support during the transition	Students' perspectives/suggestions on the pharmacist's role in helping them/supporting their transition to the Canadian healthcare system.	*"I had never used an inhaler before. Luckily, the pharmacist explained how to use it and recommended watching some YouTube videos for extra guidance."* FP21(1yr)
20	Students suggested improvements/recommendations/solutions	Includes students' suggestions/recommendations/ideas on addressing the gaps and needs to facilitate a smoother transition to the Canadian healthcare system and improve access to healthcare services.	*"I think the first thing that needs to be done is raising awareness. People come here as international students, and they have no idea how the healthcare system works."* FP8(2yr)
21	Virtual care experience	Describing the students' experiences with using virtual care services.	*"One time, I started getting a cough, so I called the clinic. I don't know whether I spoke to a doctor or a nurse, but she said it sounded like that type of cough and told me to take a COVID test and use a cough medication. I felt that it was okay because it was just a cough and I could still manage it, but if it had been something serious, I would have preferred in-person care." FP22(1yr)*
22	Pros and cons of virtual care	Including the advantages and disadvantages of using virtual care services.	*"Virtual care makes it easier to offer multilingual services for international students. It takes time for us to adapt to the language and communicate our health information accurately in English."* FP20(1yr)
23	Virtual care as a support tool	Student's perspectives and suggestions on using virtual care to support their transition to the Canadian healthcare system.	*"We can learn about the healthcare system here and how to access many services using virtual care. We can access this easily from anywhere at any time."* FP13(1yr)
24	Self-reflection	Students reflecting on what they learned about themselves.	*"I think I should have prepared more. Right now, what I need is to spend some time understanding better what the healthcare system can do for me. I feel comfortable that I can get medical attention whenever I need it, but something to improve for me would be to be more aware of what is available."* MP16(1yr)

Analytic Mapping of Codes, Themes, and Concepts

This table shows how the analysis moved from inductive codes to categories/themes and then to the four overarching concepts. Codes were not treated as mutually exclusive; therefore, a single text segment could be linked to more than one relevant code, and individual codes could contribute to more than one theme depending on the context of participants' accounts. Following the Crafting Storyline approach, themes were organized into four overarching concepts to present participants' transition experiences as a coherent storyline. The four concepts were then interpreted in relation to Transition Theory by examining how they reflected transition experiences, transition conditions, patterns of response, and movement toward adaptation.

<table>
<tr><th>Codes</th><th>Categories/Themes</th><th>Overarching Concepts</th></tr>
<tr><td>1. First experiences/impressions
2. Transition vulnerabilities/needs
3. Students personal characteristics
4. Students/families medical history</td><td>Vulnerable beginnings</td><td rowspan="3">Concept 1: Navigating the Unknown
Storyline role: Captures the beginning of the transition journey when participants encountered vulnerability and unfamiliar healthcare structures.
Transition Theory mapping: Reflects the initial transition experience and inhibiting transition conditions, including uncertainty, unmet information needs, insurance complexity, and difficulty accessing care.</td></tr>
<tr><td>1. First experiences/impressions
5. Challenges/barriers to learn about/access/use Canadian healthcare services
6. Differences between both healthcare systems
7. Potential/unverified misconceptions of the Canadian healthcare system/medical misinformation</td><td>New life, new challenges</td></tr>
<tr><td>4. Students/families medical history
5. Challenges/barriers to learn about/access/use Canadian healthcare services
6. Differences between both healthcare systems
11. Impact of challenges on everyday life/study/health</td><td>Tangled insurance and specialized care</td></tr>
<tr><td>7. Potential/unverified misconceptions of the Canadian healthcare system/medical misinformation
8. Self-educating/support
9. Family/friends support/shared knowledge/recommendations</td><td>Understanding new realities</td><td rowspan="3">Concept 2: Building Bridges
Storyline role: Shows how participants began making sense of the new healthcare system by seeking information, comparing systems, relying on support, and identifying facilitators.
Transition Theory mapping: Reflects patterns of response and facilitating transition conditions, including self-education, community knowledge, previous healthcare experiences, similarities between systems, and available supports.</td></tr>
<tr><td>10. Self-reasoning/explanations for challenges
11. Impact of challenges on everyday life/study/health
24. Self-reflection</td><td>Reflecting on challenges and consequences</td></tr>
<tr><td>12. Similarities between both healthcare systems
13. Facilitators to learn about/access/use the Canadian healthcare services</td><td>Bridging with facilitators and similarities</td></tr>
</table>

14. Similarities between both pharmacy systems<br>15. Differences between pharmacy systems in both countries<br>18. Factors impacting learning about/accessing/using pharmacy services in Canada	Common ground and contrasts in pharmacy	**Concept 3: Pharmacist as a Guide**<br>Storyline role: Positions pharmacists as accessible healthcare professionals who helped participants navigate medication use, pharmacy services, and entry points into the healthcare system.<br>Transition Theory mapping: Reflects facilitating transition conditions and supportive responses, showing how pharmacist accessibility and guidance supported participants' adaptation.
16. Pharmacy services<br>17. Pharmacy services satisfaction level<br>18. Factors impacting learning about/accessing/using pharmacy services in Canada<br>19. Pharmacist support during the transition	Pharmacy as a first resort	
16. Pharmacy services<br>17. Pharmacy services satisfaction level<br>19. Pharmacist support during the transition	Transition enhanced by pharmacists	
20. Students suggested improvements/recommendations/solutions<br>24. Self-reflection	Recommendations for a better transition journey	**Concept 4: Overcoming Barriers, Building Resilience, and Improving Access**<br>Storyline role: Represents participants' recommendations and proposed strategies to improve healthcare navigation and support future newcomer international students.<br>Transition Theory mapping: Reflects movement toward adaptation, including increased awareness, confidence, resilience, and identification of support that could improve future transitions.
18. Factors impacting learning about/accessing/using pharmacy services in Canada<br>19. Pharmacist support during the transition<br>20. Students suggested improvements/recommendations/solutions	Empowering change through pharmacist support	
20. Students suggested improvements/recommendations/solutions<br>21. Virtual care experience<br>22. Pros and cons of virtual care<br>23. Virtual care as a support tool	Bridging care gaps with virtual solutions	
